# Collagen Cross-Linking: Current Status and Future Directions

**DOI:** 10.1155/2012/406850

**Published:** 2012-01-12

**Authors:** Marine Hovakimyan, Rudolf F. Guthoff, Oliver Stachs

**Affiliations:** Department of Ophthalmology, University of Rostock, Doberaner Strasse 140, 18057 Rostock, Germany

## Abstract

Collagen cross-linking (CXL) using UVA light and riboflavin (vitamin B2) was introduced as a clinical application to stabilize the cornea by inducing cross-links within and between collagen fibers. CXL has been investigated extensively and has been shown clinically to arrest the progression of keratoconic or post-LASIK ectasia. With its minimal cost, simplicity, and proven positive clinical outcome, CXL can be regarded as a useful approach to reduce the number of penetrating keratoplasties performed. Small case series have also indicated that CXL is beneficial in corneal edema by reducing stromal swelling behavior and in keratitis by inhibiting pathogen growth. Despite these encouraging results, CXL remains a relatively new method that is potentially associated with complications. Aspects such as side effects and recurrence rates have still to be elucidated. In light of the growing interest in CXL, our paper summarizes present knowledge about this promising approach. We have intentionally endeavored to include the more relevant studies from the recent literature to provide an overview of the current status of CXL.

## 1. Introduction

Keratoconus is a noninflammatory, usually bilateral disorder, which manifests as progressive corneal instability characterized by abnormal thinning and steepening of the cornea [1]. This abnormal curvature of the cornea changes its refractive power, often resulting in irregular astigmatism and myopia and leading to mild to marked impairment in the quality of vision.

The definitive cause underlying the development of keratoconus remains unclear. However, it appears to be a heterogeneous condition that may be produced by a variety of unrelated abnormalities of a metabolic and biochemical nature. The most common presentation of keratoconus is as a sporadic disorder, in which only a significant minority of patients exhibit a family history with autosomal dominant or recessive transmission [2].

The morphological signs of keratoconus include formation of Fleischer's ring—a pigmented ring that results from the accumulation of ferritin particles in the cytoplasm of epithelial cells and widened intercellular spaces—as shown by electron microscopy [3], breaks in Bowman's membrane, filled with cells, collagen, and PAS-positive material [4], stromal thinning and abnormal keratocyte morphology [5], and endothelial polymorphism [6]. In histopathological and biochemical studies, keratoconic corneas are characterized by increased levels of proteases and other catabolic enzymes, decreased levels of tissue inhibitors of metalloproteinases (TIMPs), increased collagenolytic activity, significantly increased expression of IL-4 receptors, apoptotic cell death of keratocytes, and dramatic changes in collagen orientation and distribution [7–11].

A number of medical and surgical approaches have been used in the treatment of keratoconus. First-line treatment for patients with keratoconus is to fit rigid gas-permeable (RGP) contact lenses [12]. However, RGPs do not slow the rate of progression of the cone but merely improve visual acuity. The irregular shape of the cornea means that these lenses are challenging to fit, and the procedure requires a great deal of time and patience, with RGP fitting becoming more difficult and less successful as disease severity progresses. Moreover, owing to the formation of raised subepithelial nodular scars at or near the cone apex, contact lens intolerance can occur due to erosion and discomfort [13].

Intrastromal corneal ring segment (Intacs) implantation is a minimally invasive surgical procedure for keratoconic corneas that flattens the central corneal curvature when spectacles or contact lenses are no longer effective in improving visual acuity. The long-term tolerance of Intacs in keratoconic eyes without any significant sight-threatening complications has been reported in several studies [14, 15]. However, like contact lenses, they do not affect the corneal tissue nor do they arrest or slow keratoconus progression; instead they address the refractive consequences of the pathology by changing the shape of the cornea. The mechanical technique of tunnel creation can cause epithelial defects at the keratotomy site, anterior and posterior perforations, shallow or uneven placement of the segments, introduction of epithelial cells into the channel, and stromal thinning [16]. The femtosecond laser has been reported to reduce these complications due to more precise localization of the channel [17]. A rare but very important complication of Intacs is postimplantation infection, which may occur even many months after the initial procedure [18].

Keratoconus is one of the most common indications for keratoplasty worldwide and is in fact the leading indication in some countries [19]. Between 10% and 20% of keratoconus patients require a keratoplasty, and the procedure is increasingly indicated in the more advanced stages. The indications for keratoplasty in keratoconic patients are visual acuity below 0.5 despite optimal correction, intolerance to contact lenses, progressive corneal thinning, a decentered cone, and the presence of opacities in the visual axis. However, keratoplasty is not exempted from complications and limitations. Publications on most of the common type of keratoplasty—penetrating keratoplasty (PK)—have shown poor graft survival rates, due primarily to the continual loss of donor endothelial cells [20]. Early suture removal, as well as suture technique and graft size, has been suggested as playing a crucial role in graft failure [21]. Graft neovascularization may be a further factor associated with increased risk of graft rejection [22].

A particular problem in PK is recurrence of keratoconus caused by progressive thinning of the recipient peripheral stroma [23]. PK for keratoconus is associated with a high degree of graft astigmatism that may limit or delay visual rehabilitation. In some patients, a stable visual outcome may take several years to achieve. Another limitation of keratoplasty is the cutting of corneal nerves, leading to reduced corneal sensitivity [24].

A second surgical option for keratoconus is a deep anterior lamellar keratoplasty (DALK), involving exchange of only the epithelium and stroma. This approach has the advantage of preserving the endothelium of the recipient cornea, thus reducing risk of infection and rejection. The DALK technique involves manual dissection, which often yields an uneven bed and an irregular interface with suboptimal visual outcome. Recent improvements in surgical technique and advances in instrumentation have helped to improve the match between patient and donor corneas, leading to visual outcomes that are comparable with those after PK [25].

In recent years, the above-mentioned treatment strategies have been supplemented by collagen cross-linking using UVA light and riboflavin (vitamin B2). This is a relatively new approach, which directly targets stromal instability. Unlike all previous keratoconus management techniques, it is the only approach designed to arrest the progression of the disease. In conformity with the agreement reached at the Third Corneal Cross-Linking Congress 2007 (Zurich, Switzerland), we will use the term CXL throughout this paper to denote the combined treatment using riboflavin and UVA light.

The idea of using CXL for corneal stiffening was conceived in Germany in the 1990s [26]. The impressive clinical results initially achieved in Germany have prompted worldwide use of CXL. Currently, there are over 300 centers performing CXL in Europe, and the technique has also been used in Canada since 2008. The US Food and Drug Administration (FDA) recently approved the start of three clinical trials in the United States.

## 2. Background to Collagen Cross-Linking

Collagen I is the major macromolecular constituent of the corneal stroma, although collagen types III, V, and VI are also represented. The corneal stroma possesses the mechanical strength needed to form the anterior coat of the eye, whilst maintaining the high degree of transparency required for light transmission. Light transmission through the cornea is a result of a particular, cornea-specific arrangement of collagen fibrils. Corneal collagen uniquely forms small (32 nm), uniformly spaced fibrils that are organized into larger bundles or fibers (termed corneal lamellae) of varying thickness (1-2 *μ*m) and width (5–100 *μ*m) that are arranged in interweaving orthogonal layers throughout the stroma. They are thinner and more interwoven in the anterior cornea [27]. The characteristic collagen fibril arrangement in the cornea is believed to be maintained by the influence of different molecular subtypes within collagen fibrils and by proteoglycan macromolecules which associate in a specific manner within the collagen.

Collagen is synthesized by keratocytes in the form of its precursor molecule, called procollagen, which has two additional peptides, one at each end. In the extracellular space, specialized enzymes known as procollagen proteinases remove the two extension peptides from the ends of the molecule. The processed molecule, now referred to as collagen, undergoes posttranslational modification and begins to form fibrils and then fibers. This precisely controlled, enzyme-regulated process involves the enzyme lysyl oxidase and results in oxidation of the amino acids lysine and hydroxylysine to their respective aldehydes, which condense with other aldehydes to form intra- and intermolecular cross-links.

Another mechanism that changes the physical properties which influence the strength of the stromal tissue is nonenzymatic glycation, a phenomenon that is related to the reduced metabolic turnover of collagen and occurs through the reaction of collagen with glucose and its oxidation products. The twin findings that keratoconus progression slows with age and that keratoconus is uncommon in diabetic patients are related to collagen cross-linking via nonenzymatic glycation [28, 29].

The induction of additional cross-links is a well-established method for tissue stabilization in the polymer industries, in the preparation of prosthetic heart valves, for hardening dentistry fillings, and for tissue hardening in pathology [30]. Additional cross-link induction can be achieved by various methods, for example, nonenzymatic glycation, irradiation using UV light with or without photosensitizer, and aldehyde reactions.

The microscopic correlate of decreased corneal rigidity in keratoconus has been attributed to reductions in collagen cross-links and in molecular bonds between neighboring stromal proteoglycans. In light of this knowledge, additional cross-link induction in the corneal stroma by photopolymerization was proposed as a means of stiffening the cornea and hence of slowing disease progression.

## 3. Cross-Linking to Stabilize the Cornea

By actively increasing the degree of covalent bonding between and within the molecules of extracellular matrix, such as collagen type I and proteoglycans, therapeutic cross-linking was reasonably expected to enhance corneal rigidity and to slow or even arrest the progression of keratoconus. To investigate the possibility of cross-link induction in corneal stromal tissue, enucleated porcine eyes were divided into eight test groups (10 eyes each) that were treated with UV-light (254 nm), 0.5% riboflavin, 0.5% riboflavin and UV-light (365 nm), 5% riboflavin and blue light (436 nm), 5% riboflavin and sunlight, and the chemical agents glutaraldehyde (0.1% and 1%) and Karnovsky's solution (0.1%) [31, 32].

Measurement of biomechanical properties revealed that the greatest stiffening effect occurred after treatment with 1% glutaraldehyde. Statistically significant stiffening was also achieved in all groups except for UV-light alone and riboflavin alone.

Among all the protocols developed, the combination of UVA-light with riboflavin was selected for future experiments. The other cross-linking methods were recognized as being impractical in a clinical setting because of cytotoxicity and development of corneal haze and scarring (e.g., glutaraldehyde treatment) or application problems and prolonged treatment times (e.g., glyceraldehydes).

Riboflavin (vitamin B2) is the precursor of flavin mononucleotide (FMN) and flavin adenine dinucleotide (FAD), which are coenzymes that play a crucial role in the metabolism of proteins, fats, and carbohydrates. Riboflavin is an essential constituent of living cells and is noncytotoxic. It acts as a photomediator, considerably increasing the absorption of UVA light on exposure to corneal stoma. It has been demonstrated that absorption of UVA light within the lamellae of corneal stroma is approximately 30%, whereas combination with the photomediator properties of riboflavin increases this absorption from 30% to 95% [31, 32]. Following exposure, riboflavin is excited into a triplet state thereby generating reactive oxygen species: singlet oxygen and superoxide anions then react with available groups nearby. The precise mechanism of CXL at the molecular level has not yet been elucidated; currently, all evidence for CXL is indirect and chemical proof is awaited. One of the most plausible mechanisms of collagen cross-linking in CXL is thought to be the creation of additional chemical bonds between histidine, hydroxyproline, hydroxylysine, tyrosine, and threonine amino-acid residues. Importantly, CXL can also cause cross-linking of other classes of macromolecules within the corneal stroma, such as proteoglycans, either to one another or to collagen molecules [33].

## 4. Experimental Studies to Assess Corneal Response to CXL and Its Effectiveness

Experimental studies in rabbits have demonstrated dose-dependent keratocyte damage in a CXL procedure with surface irradiance ranging from 0.75 to 4 mW/cm^2^ [34]. At a surface irradiance of 3 mW/cm^2^ complete cell loss was observed in rabbit corneas, as shown by histology and in vivo CLSM [35, 36], whereas the same surface irradiance led to cell loss only in the anterior 250–300 *μ*m of the corneal stroma in humans [37].

Experimental studies have shown numerous Ki-67-positive fibroblasts shortly after cross-linking [35, 36], whereas only a few *α*-SMA-positive myofibroblasts were detected in the central cross-linked area [38]. Histopathological examination of human CXL-treated corneas has confirmed these findings by immunohistochemical analysis for Ki-67 and *α*-SMA [39]. This evidence indicates that the activation of keratocytes after corneal cross-linking occurs mainly by means of their transformation into fibroblasts. This may explain why no (or only mild) opacities have been observed after cross-linking in the above-mentioned studies, bearing in mind that the degree of opacity correlates directly with the number of activated keratocytes.

It is known that TGF-*β* triggers the differentiation of myofibroblasts from fibroblasts [40]. TGF-*β* is released by injured epithelial cells, and it can easily reach the adjacent stroma by penetrating the basement membrane if the latter is defective and irregular. In CXL, the epithelial sheet is removed while basement membrane integrity is retained; this feature may explain the almost complete absence of myofibroblasts and only mild (or even absent) opacification in the central treated area.

Endothelial cell density is regarded as a vitally important criterion for CXL safety. The possible cytotoxic effect of CXL has been carefully investigated in vitro in porcine endothelial cell cultures [41] and in experiments in rabbits [42], where dose-dependent endothelial cell loss was found. The latter study demonstrated necrotic and apoptotic cell death accompanied by significant edema at a standard surface UVA dose of 3 mW/cm^2^. It was shown later, however, that the endothelial monolayer had already regained its integrity 1 week after CXL, a finding that can be attributed to the proliferative capability of rabbit endothelial cells [35, 36].

It has been shown that Young's modulus and corneal rigidity are abnormally decreased in keratoconic eyes [43]. The biomechanical effect of CXL on cornea has been studied in enucleated human and porcine eyes, and stress-strain measurements were performed to evaluate changes in corneal rigidity after CXL [44]. A significant increase in corneal rigidity was shown in treated corneas, as indicated by a rise in stress. Increases in Young's modulus were also measured both in porcine and human corneas (by factors of 1.8 and 4.5, resp.). Further investigations of biomechanical properties have revealed a significantly stronger stiffening effect in anterior than in posterior stroma, both for enucleated porcine and human corneas [45]. These findings correlate positively with measurements of the thermomechanical behavior of collagen-cross-linked porcine corneas which showed a higher shrinkage temperature in the anterior stroma compared to the posterior stroma due to the higher degree of cross-linking of the anterior stroma [46].

Long-lasting improvements in the biomechanical properties of corneal tissue have been confirmed in rabbit studies for up to 8 months after CXL [47]. Morphologically, the effect of CXL has been demonstrated by measuring collagen fibril diameter in rabbit corneas [48]; transmission electron microscopy revealed a statistically significant increase in collagen fiber diameter in both anterior and posterior stroma, although this effect was more pronounced in the anterior stroma. The morphological effect of CXL has also been investigated using immunofluorescence. In one recent study, corneas with Fuchs endothelial dystrophy and bullous keratopathy from patients who had undergone CXL 7 to 90 days prior to penetrating keratoplasty were investigated by immunofluorescence for collagen type I and compared with control groups with same disorders [49]. Cross-linked corneas showed a fluorescent anterior lamellar zone with condensed and highly organized collagen fibers, which was not the case in the non-cross-linked group. The intensity of fluorescent staining was shown to decrease gradually in an anterior-posterior direction. It should be also noted that the same study demonstrated a diminution of the cross-linking effect over time.

CXL has been reported to have a certain impact on the biochemical properties of corneal tissue by increasing the resistance of corneal matrix against digestion by proteolytic enzymes such as pepsin, trypsin, and collagenase [50]. The authors attribute the stabilizing biochemical effect of CXL to alterations in the tertiary structure of collagen fibrils, thus denying the proteolytic enzymes access to their target sites.

It has been proposed that a positive correlation exists between corneal stiffness and intraocular pressure (IOP) [51]. On the basis of this correlation, an increase in IOP can be expected following CXL as indirect confirmation of the efficacy of cross-linking. In fact, IOP increases have been recorded in patients after CXL, reflecting increased corneal rigidity [52–54].

Two-photon microscopy has been used in rabbit corneas to visualize cross-linking effects on cells and collagen after CXL by detecting second harmonic generation (SHG) and autofluorescence [55]. In this study, the grade of cross-linking was quantified by autofluorescence lifetime measurements. CXL effects were detected for up to 2 weeks in the anterior stroma. Future experiments with longer follow-up are required to determine the stability of these effects.

## 5. Corneal Response to CXL In Vivo

Alongside histological studies in animals, in vivo confocal laser-scanning microscopy (CLSM) now provides a noninvasive modality for observing the corneal response to CXL. In vivo CLSM offers obvious advantages as a noninvasive imaging technique that permits dynamic investigations to be conducted over long observation periods [56]. Importantly, in vivo CLSM not only provides an overview of the tissues but also allows quantification of acquired images, thus yielding quantitative as well as qualitative data, and this is a key aspect when evaluating the safety and duration of the effects achieved. The first results obtained with in vivo CLSM after CXL were published in 2006 [57]. This study demonstrated epithelial regeneration and normal morphology as early as day 5 after CXL. Subepithelial stromal nerve fibers were noted to have disappeared immediately after CXL: initial signs of regeneration were observed 1 month after the operation and continued throughout the postoperative period, with normal morphology and corneal sensitivity being restored after 6 months. In the same patients, disappearance of keratocytes in the anterior stroma and rarefaction in the posterior stroma were reported soon after treatment [58]. In good agreement with previous experimental studies, a repopulation process was demonstrated, revealing activated keratocytes in the anterior and intermediate stroma. Normal endothelial morphology and density were observed by in vivo CLSM during the entire follow-up period. These findings were confirmed in a larger patient cohort that was followed using in vivo CLSM for up to 3 years [59]. Interestingly, this study with a longer follow-up showed that, despite complete nerve fiber regeneration at 6 months postoperatively, the plexus structure could not be defined until 1 year after CXL. A later in vivo study with CLSM, again with 3-year follow-up, confirmed these findings [60]. In this study, occasional inflammatory cells were present in the different epithelial layers 1 month after CXL. Importantly, this had never been reported previously in patients, whereas experimental animal studies had revealed inflammatory cell activation in corneal stroma after CXL [35, 61]. Notably, the authors also showed that the degree of stromal changes observed after the first week varied among patients and was unrelated to the severity of keratoconus. Using in vivo CLSM, Kymionis et al. [62] have shown that tissue alterations after CXL are quite similar in patients with keratoconus and post-LASIK corneal ectasia.

In vivo CSLM has revealed early and late demarcation lines between treated and untreated stroma. Demarcation lines have also been detected by slit-lamp examination [63] and anterior segment optical coherence tomography [64]. All the aforementioned in vivo CLSM studies have yielded relatively similar results regarding the cellular and extracellular changes after CXL. Although in vivo CLSM is an indispensable tool for the dynamic sequential study of wound healing after CXL, the technique is limited in that it depicts only small areas (300 *μ*m) and image interpretation is based on morphological features and reflectivity. It is still strongly recommended that every excised cornea should be investigated by electron microscopy and immunohistochemistry.

## 6. Clinical Results

Widely accepted parameters for evaluating the clinical outcome of refractive corrections and CXL include uncorrected visual acuity (UCVA), best corrected visual acuity (BCVA), uncorrected distance visual acuity (UDVA), corrected distance visual acuity (CDVA), apex curvature, and topography-derived outcomes of maximum and average keratometry values.

A prospective, nonrandomized pilot study published in 2003 reported the earliest clinical experiences in a series of 23 eyes with moderate or advanced progressive keratoconus [65]. During follow-up, which lasted for between 3 months and 4 years, not only disease progression was at least halted, but, in 70% cases, there was also a statistically significant improvement in BCVA, correlating with a reduction of the maximal keratometry readings by 2 diopters and of the refractive error by 1.14 diopters.

In an uncontrolled retrospective study, Raiskup-Wolf et al. [66] showed that the flattening process continues over a period of years: they followed a large cohort of patients (480 eyes of 272 patients) for up to 6 years and reported arrested keratoconus progression and significant improvements in visual acuity. The long-term stabilization of keratoconic corneas without significant side-effects has also been demonstrated in 44 eyes for up to 48 months after CXL [67], also accompanied by a reduction in the mean *K* value by 2 diopters and gradually increasing improvements in UCVA and BCVA during the observation period. The statistical significance of these values was maintained after 36 and 48 months of follow up.

Vinciguerra et al. [68] have described the outcome of CXL in 28 eyes with progressive keratoconus, using the fellow eye as control. This nonrandomized study revealed a statistically significant improvement in UCVA and BCVA, with a reduction in the steepest simulated keratometry meridian by as much as 6.16 diopters. Keratoconus in the untreated fellow eye showed progression over the same period.

In light of experimental studies reporting increased corneal stiffness after CXL, efforts have also been undertaken to evaluate biomechanical parameters such as corneal hysteresis and corneal resistance factor after CXL [69, 70]. Using the Ocular Response Analyzer, both studies revealed only slight, statistically nonsignificant changes in biomechanical parameters. This inconsistency has been attributed to the different methodologies used and to inherent differences between in vitro and in vivo models. Comparisons between treated and untreated eyes of the same patients, performed over a 1-year period following CXL, showed significant flattening and hence decreases in keratoconus indices in the CXL-treated cornea; these findings were seen as indicating a shift toward a more regular corneal shape, whereas the same parameters in the control eyes indicated disease progression [71]. All these studies have concluded that the improvement in vision after CXL is produced by a decrease in astigmatism and corneal curvature as well as by an increase in corneal rigidity, leading to topographical homogenization.

Despite the positive outcomes reported from nonrandomized clinical studies, these findings are still preliminary and should be interpreted with caution. It is clear that conclusive evidence regarding the effects of CXL will emerge only from multiple randomized controlled trials (RCTs) with a more robust design, and, to date, the literature on CXL includes only a very small number of such RCTs. In the Melbourne study, which was conducted in 49 patients, statistically significant differences were observed between control and treatment groups in terms of BCVA and *K* values for up to 12 months after CXL [72]. More recently, another group has published an RCT reporting on one-year results after CXL for the treatment of keratoconus and corneal ectasia [73]. CXL was shown to be effective in improving UDVA, CDVA, the maximum *K* value, and the average *K* value. Keratoconus patients displayed greater improvement in topographic measurements than patients with corneal ectasia.

Combination of CXL with other treatment modalities also appears to be useful for enhancing clinical outcomes in keratoconus management. The combination of topography-guided PRK with CXL is reported to be an optimal method for attaining greater effects in progressive keratoconus [74, 75], as reflected in rapid and significant improvements in visual acuity (both UCVA and BCVA) and marked correction of corneal irregularities, as shown by topographic evaluation. Comparison of sequential versus same-day simultaneous CXL and topography-guided PRK demonstrated the greater impact of the same-day procedure in reducing *K* values, improving visual acuity, and lessening corneal haze [76]. Furthermore, the addition of CXL to Intacs insertion has resulted in significantly greater clinical improvements in keratoconus than Intacs insertion alone [77]. However, these data are preliminary, and further confirmation is required from future studies.

## 7. Alternative Strategies

Although CXL has proved itself in clinical practice, efforts are still being made to modify the standard protocol to increase patient safety and comfort. Two factors in particular are critical to ensure the success of CXL therapy: a certain minimum corneal thickness prior to CXL and the guaranteed presence of a specific concentration of riboflavin. The standard protocol for CXL uses 0.1% riboflavin in 20% dextran.

Quite recently, a novel protocol using hypoosmolar riboflavin solution has been developed for the management of thin corneas [78]. The hypoosmolar solution achieved preoperative swelling of thin corneas, thus enabling CXL to be undertaken and keratoconus progression to be arrested in all cases treated. It should be mentioned, however, that all corneas included had a minimum stromal thickness of 323 *μ*m. Importantly, CXL failure has been reported in an extremely thin cornea, suggesting that a minimum preoperative stromal thickness of 330 *μ*m is needed for successful CXL [79].

The widely accepted standard protocol for CXL involves corneal epithelial debridement to facilitate the penetration of riboflavin into the stroma. However, deepithelialization is accompanied by pain, foreign body sensation, and discomfort in the form of burning and tearing for many days. Moreover, epithelial debridement reduces total corneal thickness, and this can have dramatic repercussions in extremely thin corneas. Researchers and clinicians have therefore been motivated to develop a variant of the standard CXL technique without deepithelialization, thus offering patients safer and faster CXL while retaining the efficacy of the standard approach.

An immunofluorescence confocal imaging study on enucleated porcine eyes has shown that, without previous deepithelialization, CXL had no effect on collagen type I organization, confirming the belief that the intact epithelium acts as barrier to riboflavin absorption [80]. The necessity for deepithelialization as an initial step in CXL has also been demonstrated in vitro on porcine eyes by other investigators [81, 82]. Analysis of corneal light transmission spectra in these studies clearly revealed that riboflavin was able to penetrate into the stroma only in completely abraded corneas.

In one clinical study, deepithelialization was customized on the basis of pachymetric measurements so that the cornea was left intact in regions thinner than 400 *μ*m and removed in regions with adequate thickness [83]. This study demonstrated an increase in the safety of CXL, as well as satisfactory results in stabilizing keratoconus, but without detailing special effects and alterations in specific areas. To evaluate intrastromal concentrations of riboflavin in CXL with and without epithelial debridement, half of the corneas (from keratoconus patients enrolled for keratoplasty) underwent CXL with abrasion and half without [84]. Quantitative HPLC analysis demonstrated that a theoretically safe and effective concentration of riboflavin was obtained only after epithelial debridement, confirming previous results. In a similar comparative study, customized deepithelialization was performed in two keratoconus patients, and anterior segment OCT and in vivo CLSM were used to compare CXL effects in epithelium-on and epithelium-off regions of the same cornea [85]. Both techniques detected strong CXL effects (e.g., the demarcation line and keratocyte disappearance followed by haze) in the deepithelialized regions, whereas the corneal stroma under the intact epithelium seemed to be spared.

Another morphological modification of the CXL protocol without deepithelialization proposes the use of benzalkonium chloride (BAC) [86], given its ability to increase epithelial permeability by loosening tight junctions [87]. In an experimental comparative study, CXL incorporating application of 0.005% BAC was performed on rabbit corneas with intact epithelium [88]. The biomechanical stiffening effect was reduced by about one-fifth compared with standard CXL. Increasing the BAC concentration further to 0.02% clearly affected epithelial permeability to riboflavin, thus increasing the absorption coefficient and achieving greater stiffening effects, as reflected in a significant increase in Young's modulus and stress-strain values [89]. Moreover, study of resistance to enzymatic digestion revealed that corneas with intact epithelium treated with CXL without BAC dissolved in collagenase solution after 6 days, whereas corneas with intact epithelium undergoing CXL with BAC behaved like controls (standard CXL protocol) and did not dissolve even after 14 days.

In a prospective, consecutive clinical study in patients with progressive keratoconus, CXL with intact epithelium and pretreatment with BAC, and other substances enhancing epithelial permeability revealed less pronounced effects than standard CXL [90]. Despite a positive effect on CDVA, corneal curvature was maintained and did not improve.

The long-term efficacy and possible side effects of CXL without deepithelialization require further assessment in randomized, controlled studies with longer observation periods.

## 8. Other Applications of CXL

As the technique has grown in popularity in recent years, other potential applications for CXL in ophthalmology have been proposed.

### 8.1. Ectasia

Ectasia following corneal excimer laser refractive surgery is a postoperative thinning of the cornea, encountered mainly after LASIK. In the most advanced cases, penetrating keratoplasty may even be required. Thanks to its ability to stabilize the biomechanics of the cornea, CXL has been proposed as a therapeutic method of arresting keratectasia progression. The first study to investigate the advantages of CXL in post-LASIK ectasia showed an increase in biomechanical stability sufficient to prevent the progression of keratectasia [91]. A reduction in maximum keratometric readings and an increase in biomechanical stability were also confirmed in a larger group (10 patients) for up to 12 months [92]. CXL has been reported to improve BSCVA in eyes with post-LASIK ectasia without any significant side effects [93]. In vivo CLSM disclosed relatively similar corneal alterations after CXL both in keratoconic and in post-LASIK corneal ectasia eyes [62]. The positive impact of CXL in conjunction with PRK has been shown in a case of post-LASIK ectasia [94]. As a qualifying remark, it should be pointed out that the longest follow-up in these studies has been 1 year. Despite these promising early results, studies with longer follow-up and larger patient numbers are needed to evaluate the effectiveness and safety of CXL in the management of post-LASIK keratectasia.

Another form of corneal ectasia is pellucid marginal degeneration (PMD)—a noninflammatory disorder characterized by a peripheral band of thinning of the corneal segment between the 4 o'clock and 8 o'clock positions. One case report describes a patient with bilateral PMD who underwent CXL unilaterally, on the side with more profound ectasia [95]. The treated eye was examined during the first year after treatment. Reduced corneal astigmatism and improved visual acuity were detected at 3 months and remained stable through the 12-month interval. The endothelial cell count and corneal thickness remained unchanged from the preoperative assessment to 12 months, confirming the safety of CXL. A significant positive clinical outcome has also been obtained in PMD patients by combining CXL with photorefractive keratectomy [96] or with topography-guided transepithelial surface ablation [97].

### 8.2. Keratitis

Keratitis is a corneal disease that may result from infection (with bacteria, fungi, yeasts, viruses, and amoebae) or from immunological disorders (sterile keratitis). Attempts have been made in recent years to assess the effects of CXL in infectious keratitis, with a possible view to making it a treatment option. The antibacterial effectiveness of CXL has been demonstrated in vitro against some common pathogens, selected from a panel of clinical ocular isolates obtained from patients with severe bacterial keratitis [98]. CXL has also exhibited antimicrobial effects in vitro against fungal pathogens, such as *Candida albicans*, *Fusarium* sp., and *Aspergillus fumigatus* [99]. The ability of CXL to inhibit pathogen growth has the potential to make it an effective tool in the management of infectious keratitis. In a small case series with bacterial and fungal keratitis, Iseli et al. [100] demonstrated immediate regression of the corneal melting process and a decrease in infiltrate size after CXL in most patients. A later study has confirmed this observation and demonstrated symptomatic relief and arrest of melting progression in corneas with bacterial keratitis [101].

CXL has also been beneficial in treating a patient with *Escherichia coli* keratitis [102], leading to complete healing of corneal ulceration, regression of edema, and disappearance of painful symptoms.

The antimicrobial effects of CXL have two possible mechanisms, and, in all probabilities, these operate in synergy. Firstly, the pathogens implicated in corneal melting are known to act by enzymatic digestion. Since CXL has been reported to increase tissue resistance to enzymatic digestion [50], the improvement in symptoms can be attributed to the greater resistance of corneal tissue to enzyme activity. Secondly, the phenomenon of cell apoptosis after CXL may include not only keratocytes but also pathogens, thus arresting the infectious process.

Despite these promising results, both studies referred to above were limited in terms of case numbers, and further extensive investigations are needed before CXL can be incorporated into routine clinical practice for the treatment of keratitis.

### 8.3. Corneal Edema

Endothelial dysfunction results in corneal swelling (edema) and visual impairment. The most common conditions associated with edema are bullous keratopathy and Fuchs dystrophy. In an experimental study, Wollensak et al. [103] demonstrated in enucleated porcine eyes that the CXL procedure altered the swelling pattern of the cornea, minimizing hydration. It is hypothesized that CXL strengthens the interfiber attachments, thereby reducing the space for fluid accumulation and so increasing the optical clarity of the cornea. The antiedematous effect of CXL potentially makes it a useful tool in the clinic for treating corneal edema. Reduced corneal edema, increased corneal clarity, and improved visual acuity were demonstrated during the 2-month follow-up period after CXL in two cases of bullous keratopathy associated with corneal ulcer or infectious keratitis [104]. CXL performed in eyes with endothelial decompensation and nonhealing ulcers led to significant benefit in only half of nonhealing ulcer cases, whereas a significant decrease in corneal thickness and improvement in symptoms was noted in 10 of 11 corneas with endothelial decompensation for up to 3 months after treatment [105]. However, in another clinical study where CXL was performed in patients with bullous keratopathy and Fuchs dystrophy, measurements of central corneal thickness for up to 6 months indicated that CXL reduced edema only temporarily [106]. Moreover, in advanced cases of corneal edema, CXL had only little effect, suggesting that the greater stromal impairment in these patients interfered with riboflavin penetration, thus resulting in less benefit. On the other hand, stable reductions in corneal thickness have been demonstrated in bullous keratopathy and Fuchs dystrophy for up to 8 months after CXL [107]. This inconsistency can be explained by the fact that Wollensak et al. [107] used pretreatment with 40% glucose for 1 day prior to CXL, allowing dehydration of the cornea and more precise estimation of corneal thickness. The importance of dehydrating the swollen cornea prior to CXL has also been demonstrated by another group in Fuchs dystrophy patients with various degrees of edema [108]. Although the results reported by Wollensak et al. [107] appear to be more reliable, their study included only a very limited number of patients. Future studies with longer follow-up and larger patient numbers are necessary to assess the efficacy and limitations of CXL in the treatment of corneal edema. At present, as a minimally invasive and safe procedure, CXL can be recommended at least for those patients awaiting keratoplasty.

## 9. Complications after CXL

Although CXL is one of the most promising developments in the management of keratoconus, the potential for adverse outcomes should not be underestimated.

### 9.1. Stromal Haze

Corneal scarring (diffuse subepithelial opacification) that was slow to resolve has been reported in a 41-year-old man after CXL; this complication responded to topical application of steroids, eventually resolving gradually after several months [109]. Subclinical stromal haze that does not impair patients' vision has also been detected by other groups using confocal microscopy [59, 110]. Raiskup et al. [111] demonstrated CXL-induced permanent corneal haze in approximately 8.6% of all treated eyes. The haze of varying degrees after CXL took up to 12 months to resolve completely [66].

Interestingly, haze formation correlates positively in all studies with the stage of keratoconus; all patients who developed haze had advanced keratoconus, thinner corneas, Vogt's striae, and higher keratometry values. Consequently, the development of haze may be attributed not to CXL itself but to the stage of keratoconus. Objective quantification of the time course of CXL-induced haze suggests that it peaks at 1 month, plateaus at 3 months, and then gradually decreases thereafter [112].

More recently, corneal haze formation has been reported in the posterior stroma in 2 patients (7% of all treated cases) after CXL [113]. Notably, both patients had mild rather than advanced keratoconus. Even though haze developed near the apex of the cone, away from the central visual axis, these cases indicate that not only keratoconus stage but other factors too may have contributed to this phenomenon. Further research is needed to investigate this aspect carefully with regard to the safety of CXL.

A far higher incidence of haze has been observed in 46.42% of keratoconic eyes treated with simultaneous customized PRK and CXL [114]. Posterior linear stromal haze was visualized clinically by slit-lamp biomicroscopy, and, in the same corneas, in vivo CLSM revealed highly reflective, spindle-shaped keratocytes, which are associated with stromal repopulation and increased collagen deposition. Photoablation is highly likely to have been the factor that contributed to the more common occurrence of haze with this combined approach.

### 9.2. Keratitis

There is no direct evidence yet of any lowering of the corneal immune mechanisms following CXL. Nevertheless, there is a major risk of infection after standard CXL because the procedure involves deepithelialization followed by the application of a soft contact lens. There has been one case report of polymicrobial keratitis caused by *Streptococcus salivarius*, *Streptococcus oralis*, and *Staphylococcus* sp. 3 days after CXL [115]. The patient admitted that he had cleaned his bandage contact lens in his mouth, an action that was most probably the cause of keratitis. Escherichia coli infection occurred in a case 3 days after CXL, with multiple stromal infiltrations and moderate anterior chamber inflammation [116]. The bacterial keratitis was successfully treated with fortified tobramycin and cephazolin eye drops for several weeks. Furthermore, fungal keratitis has been reported as a complication 22 days after CXL despite complete epithelial recovery at day 5 [117]. Koppen et al. [118] have also published a case report series concerning 4 patients who developed severe keratitis, resulting in corneal scarring within the first few days after CXL.

Acanthamoeba keratitis with corneal melting has been reported in a 32-year-old woman 5 days following CXL [119]. The patient was unaware of wearing a bandage contact lens and repeatedly rinsed his face and eyelids with tap water. Because of corneal perforation, a large therapeutic keratoplasty à chaud was performed.

A case report has shown that CXL can induce herpetic keratitis with iritis even in patients with no history of herpetic disease [120]. Following steroids and acyclovir treatment, a significant improvement was observed, and there was no evidence of herpetic disease recurrence 2 months postoperatively. The same group reported on the diffuse lamellar keratitis during the first posttreatment days [121], which resolved after intensive treatment with topical corticosteroids during the following 2 weeks.

More recently, 2 cases of keratopathy have been reported after uneventful CXL for grade 3 keratoconus [122]. The pathogenesis in these cases remained unidentified. Corneal infiltrates slowly resolved after combined topical antibiotic/antifungal/steroids treatment.

Although the incidence of keratitis is low in these studies, it remains a very serious side effect of CXL.

## 10. Concluding Remarks

CXL, a procedure that uses UVA light in conjunction with riboflavin as a photomediator, creates new covalent cross-links between collagen fibrils, thus strengthening and stabilizing the cornea. CXL is a topic that has been attracting growing interest over the past decade. It has been shown not only to arrest progression of keratectasia in progressive keratoconus and post-LASIK corneas but also to exert a moderately positive effect on visual status. Corneal edema and infectious keratitis have also been reported to benefit from CXL. Because most of the published clinical findings have come from nonrandomized studies, further corroboration is required from more robustly designed RCTs.

Attempts have been made to optimize CXL to minimize the potential for risk in very thin corneas or to reduce patient discomfort. The major safety concerns associated with CXL are ocular surface damage and endothelial cell damage. Although the safety of CXL has been demonstrated in numerous experimental animal studies, outcomes in patients are more complex than in animal models. Postoperative events vary markedly, depending on the stage of keratoconus in patients treated. Edema, stromal haze, and infectious keratitis have been reported as complications in the clinical setting-albeit very rarely. Further studies are therefore needed to extensively investigate the safety of the CXL procedure.

Because collagen turnover in the stroma is known to take several years, it remains unclear whether the changes in corneal stability reported after CXL will be permanent or whether its effects are time limited. The long-term effects of standard and modified protocols for CXL should be reviewed thoroughly in studies with longer follow-up.
